# Genetic testing can resolve diagnostic confusion in Alport syndrome

**DOI:** 10.1093/ckj/sft144

**Published:** 2013-12-18

**Authors:** Jennifer Adam, Thomas M. F. Connor, Katrina Wood, David Lewis, Ramesh Naik, Daniel P. Gale, John A. Sayer

**Affiliations:** 1 Newcastle Hospitals NHS Foundation Trust, Newcastle upon Tyne, UK; 2 West London Renal and Transplant Centre, Hammersmith Hospital, London, UK; 3 The Oxford Kidney Unit, Churchill Hospital, Oxford, UK; 4 Royal Berkshire Hospital, Reading, UK; 5 UCL Centre for Nephrology, Royal Free Hospital, London, UK; 6 Institute of Genetic Medicine, Newcastle University, International Centre for Life, Newcastle upon Tyne, UK

**Keywords:** Alport syndrome, COL4A3, COL4A5, haematuria, molecular genetics

## Abstract

Alport syndrome (AS) is a familial glomerular disorder resulting from mutations in the genes encoding several members of the type IV collagen protein family. Despite advances in molecular genetics, renal biopsy remains an important initial diagnostic tool. Histological diagnosis is challenging as features may be non-specific, particularly early in the disease course and in females with X-linked disease. We present three families for whom there was difficulty in correctly diagnosing AS or thin basement membrane nephropathy as a result of misinterpretation of non-specific and incomplete histology. We highlight the importance of electron microscopy and immunofluorescence in improving diagnostic yield and also the hazard of interpreting a descriptive histological term as a diagnostic label. Molecular genetic testing allows a definitive diagnosis to be made in index patients and at-risk family members.

## Introduction

Alport syndrome (AS) is an inherited disease that results in progressive glomerular damage, often in association with sensorineural hearing loss and ocular disease. It is caused by a number of genetic mutations affecting type IV collagen, a constituent of the glomerular basement membrane, cochlea and eye [1–3]. Type IV collagen is a heterotrimeric extracellular protein that forms an important part of the glomerular basement membrane. In the adult kidney this complex is composed of subunits encoded by the genes *COL4A3* and *COL4A4* (which are situated on chromosome 2) and *COL4A5* which lies on the X chromosome. AS may be caused by homozygous or compound heterozygous mutations of *COL4A3* or *COL4A4*, or by hemizygosity for a single defective *COL4A5* allele in males [4]. This explains the autosomal-recessive and X-linked modes of inheritance that are seen in clinical practice. Heterozygosity for a pathogenic mutation of any of these genes can be associated with microscopic haematuria, thinning of the glomerular basement membranes and a low, but non-negligible, risk of renal impairment. Known heterozygous carriers of AS therefore require long-term medical follow-up [4].

Advances in molecular genetics, including the discovery of the *COL4A* family of genes, have led to the use of genetic testing in confirming a diagnosis of AS. Detection of mutations in *COL4A5* gene in males with X-linked AS can be up to 95% using contemporary techniques [5]. However, in patients with suggestive clinical features and or a relevant family history, renal biopsy remains the primary initial diagnostic tool used to identify patients with AS [4].

Diagnosing AS histologically can be challenging because findings on light microscopy are often non-specific. Classically, patients exhibit increased glomerular cellularity and an interstitial infiltrate containing lipid-laden foam cells, which is a bad prognostic marker [6]. However, patients may also exhibit glomerulosclerosis and mesangial changes which can be misleading. Hallmark ultrastructural lesions such as thinning, thickening and longitudinal splitting of the glomerular basement membrane strongly suggest type IV collagen-associated disease but cannot always distinguish AS from thin glomerular basement nephropathy (TBMN) [7]. In addition, electron microscopy (EM) of glomeruli is not always available. Here we present three families in which incomplete or incorrectly interpreted clinical and histological data led to diagnostic inaccuracies that were resolved by genetic testing.

## Case reports

### Family 1

**Fig. 1.**
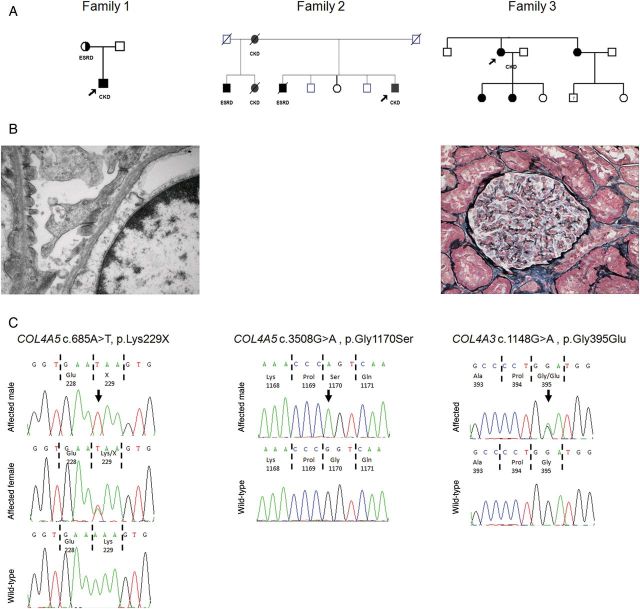
Family structure, genetic analysis and histological features. (**A**) Pedigree structure of Family 1, 2 and 3. Probands are arrowed. Squares represent males, circles represent females. Affected individuals are shaded. CKD, chronic kidney disease; ESRD, end-stage renal disease. (**B**) Left, renal biopsy EM image from proband of Family 1 showing thinning of the glomerular basement membrane. Right, renal biopsy under light microscopy (silver stain) of proband from Family 3 revealed only very mild mesangial proliferative changes. (**C**) Sequence chromatograms of *COL4A5* (Family 1 and 2) and *COL4A3* (Family 3) in affected patients with wild-type control. Nucleotides are shown and corresponding amino acids are numbered. Sequence variants are arrowed.

A 26-year-old man presented with hypertension, haematuria, nephrotic-range proteinuria and a normal GFR. Of note, his past medical history included sensorineural deafness and his mother also had a history of chronic kidney disease (Figure 1A).

He underwent a renal biopsy which revealed on light microscopy features consistent with focal segmental glomerulosclerosis (FSGS) with hypertensive changes. However electron microscopy (EM) examination of the biopsy revealed splitting of the glomerular basement membrane, consistent with AS (Figure 1B).

His mother had reached end-stage renal disease (ESRD) at 32 years of age and had undergone a successful renal transplant. She carried the diagnostic label of membranoproliferative glomerulonephritis (MPGN). A review of her native renal biopsy revealed the changes were limited to mild mesangial proliferation on light microscopy. Unfortunately there were no glomeruli in EM or immunofluorescence (IMF) specimens to allow elaboration of these findings. These features on light microscopy had, over time, been mistranslated into a diagnostic label of MPGN.

Genetic testing confirmed that both mother and son had a novel c.685A>T, p.Lys229X mutation in the *COL4A5* gene (Figure 1C), which is predicted to be pathogenic, confirming X-linked AS.

The proband developed progressive renal impairment associated with persistent proteinuria. Had his mother's initial diagnosis been accurate, his renal biopsy could have been avoided and earlier detection and management of his hypertension and proteinuria may have slowed the rate of decline of his renal function.

### Family 2

A 25-year-old man presented in 1984 with intermittent macroscopic haematuria, hypertension, normal renal function (creatinine clearance 154 mL/min) and proteinuria (0.2 g/24 h). His renal function has remained stable over 29 years of follow-up (serum creatinine 93 µmol/L aged 54 years). A family history revealed two brothers who progressed to ESRD aged 46 and 57, as well as a sister and mother affected with chronic kidney disease in whom no histological or clinical diagnosis was available (Figure 1A).

The proband underwent renal biopsy in 1985 which showed possible mesangial expansion with no significant immunoperoxidase staining, and was interpreted as ‘possible MPGN’. His half-brother was biopsied in 1987, showing glomerulosclerosis associated with a proliferative glomerulonephritis. His elder brother was biopsied in 1996 when he too had advanced renal impairment (serum creatinine 390 µmol/L), demonstrating advanced glomerulosclerosis with mesangial expansion but negative immunofluorescence. There were no EM reports or photos from the 1985 biopsy, and the 1996 biopsy had no glomeruli in the tissue submitted for EM.

DNA analysis using whole exome sequencing confirmed that the proband carried the c.G3508A mutation in *COL4A5* (Figure 1C), which is known to cause X-linked AS [8]. This mutation was also found in his half-brother on dialysis but not in his unaffected brothers. The proband's affected brother had died 7 years prior to the identification of this mutation. We were however able to perform PCR on DNA extracted from serum stored at the transplant unit and confirmed that he too had the identical mutation.

There was no family history of deafness; however, subsequent auditory assessment in the index case revealed mild left-sided presbyacusis with more marked hearing loss down to 60 dB on the right. There were also no characteristic lens abnormalities; however, there were peripheral retinal changes that might be associated with AS.

### Family 3

A 47-year-old woman presented with haematuria, hypertension, abnormal renal function (serum creatinine 114 µmol/L), proteinuria and mild high-tone sensorineural hearing loss. A family history revealed a sister with hearing loss and microscopic haematuria, a nephew with microscopic haematuria and two daughters with microscopic haematuria, suggesting a dominant inheritance pattern (Figure 1A). A renal biopsy of the proband showed mild mesangial proliferative changes (Figure 1B) but no specific diagnostic features.

The biopsy was insufficient to allow EM analysis. The association of high-frequency hearing loss and haematuria raised the suspicion of a hereditary nephritis.

DNA analysis confirmed that the proband, her sister and two of her three daughters were heterozygotes for a novel pathogenic mutation of the COL4A3 gene (c.1148G.A, p.Gly395Glu) (Figure 1C). This indicates a diagnosis of autosomal-dominant TBMN. This variant is predicted to interrupt the GLY-X-Y repeat structure of the collagenous domain of the alpha-3 chain. Missense changes within this region have been previously reported in AS patients [9]. These family members now are now under regular renal follow-up and fortunately have only microscopic haematuria with normal renal function and blood pressure at present.

## Discussion

The difficulty in securing a diagnosis of AS from histological analysis of renal biopsy specimens has been described in a Chinese case–control study by Yao *et al.* [10]. The authors revealed that mesangial proliferative glomerulonephritis was the most common misdiagnosis in male patients with X-linked AS and that FSGS was the most common misdiagnosis in female patients with X-linked AS. It was acknowledged that often EM and collagen protein immunofluorescence studies were not available or had not been performed on biopsy samples from those misdiagnosed.

Females heterozygous for mutations in *COL4A5* that cause X-linked AS have a variable phenotype, with ∼15% eventually developing ESRD, at a median age of 49 years—a risk broadly similar to that seen in individuals with AD TBMN who are heterozygous for pathogenic autosomal *COL4A3* and *COL4A4* mutations [11, 12]. This is in marked contrast to males with X-linked AS (who are hemizygous for pathogenic *COL4A5* mutations) or individuals homozygous or compound heterozygous for autosomal *COL4A3* or *COL4A4* mutations, in whom the risk of ESRD is >90%, with median age of onset 25 years or less [13, 14].

Analysis of the pedigree of *Family 1* presented here suggests that the proband's mother is likely to have had a *de novo* mutation. Both of these factors may have conspired to make AS a less likely clinical differential at the time of her original biopsy. Coupled with the non-specific findings on histology and in the absence of information from EM and IMF studies, her misdiagnosis seems almost inevitable. However, we note that a review of this biopsy concluded that although some glomeruli exhibited mesangial proliferative changes, these were not sufficient to warrant a histological diagnosis of MPGN. This case demonstrates that misinterpretation and mistranslation of a descriptive into a diagnostic term can be a crucial flaw leading to misdiagnosis. It is conceivable that a proportion of misdiagnoses identified by Yao *et al.* may have been related to a similar error in interpretation.

In Family 2, the relatively mild disease manifestations in the proband, coupled with insufficient or unavailable biopsy material for electron microscopic examination, had also led to the histological appearances of mesangial proliferation being translated into a diagnostic label of possible MPGN. Molecular testing allowed the correct diagnosis of X-linked AS to be made.

In Family 3 an original clinical diagnosis of probable X-linked AS was revised by the identification of a pathogenic heterozygous autosomal *COL4A3* mutation co-segregating with disease. This molecular diagnosis has had significant implications for the family, since it is now clear that male offspring of affected female members of this family bear a 50% risk of inheriting TBMN and are not at risk of the more severe X-linked AS.

Extra-renal features of AS, including sensorineural deafness, anterior lenticonus and dot and fleck retinopathy can provide important diagnostic clues. However, as illustrated here and previously, these features are not always present even when there is advanced renal disease [15, 16]. In addition, sensorineural hearing impairment is sometimes seen in TBMN and other disorders that cause kidney failure, such as autosomal-dominant *MYH9*-associated diseases, including Fechtner and Epstein syndromes [17] and branchio-oto-renal syndrome, which is caused by heterozygous mutations of the *EYA1, SIX1* and *SIX5* genes [18]. While extra-renal features may be helpful in suggesting a likely clinical differential diagnosis, they cannot be relied upon to make a firm diagnosis in familial kidney diseases.

In order to accurately identify families with AS, care must be taken by nephrologists in interpreting non-specific histological terminology, and vigilance in separating descriptive histological terms from diagnostic labels is needed. Furthermore, it is vital to ensure EM and ideally IF for collagen proteins are available and reviewed in patients with unexplained haematuria even in the absence of a family history. Light microscopic appearances of proliferative glomerulonephritis can also be caused by non-immune-mediated diseases, including type IV collagen abnormalities, which should feature in the differential diagnosis unless there is good evidence of immune-complex disease on clinical, serological, immunostaining or ultrastructural grounds.

Although not 100% sensitive, genetic testing can provide a definitive means of making a diagnosis of AS in the vast majority of cases and (in the absence of male-to-male transmission elsewhere in the family) is the most reliable way to distinguish females who are carriers of X-linked AS from those with heterozygous *COL4A3* or *COL4A4* mutations. This distinction may be especially important in women planning to start a family, since in the former situation male, but not female, offspring will have a significant risk of ESRD in childhood and this knowledge may inform a decision to undergo prenatal testing. Once a genetic diagnosis has been made, this can enable screening of symptomatic or at-risk family members and may obviate the need for a kidney biopsy in some circumstances.

In addition to diagnostic information, uncovering the genetic change in a patient with AS may provide additional prognostic information, since early stop codons, frameshift mutations, large deletions and rearrangements have been associated with earlier onset renal failure and deafness than missense mutations and these more destructive genetic alterations are possibly more likely to be associated with *de novo* antiglomerular basement membrane disease following transplantation [13]. Together these cases illustrate the diagnostic power that the application of molecular genetics to clinical practice can bring to the diagnosis of patients with kidney disease.

## Conflict of interest statement

None declared.
